# SLC26A11 (KBAT) in Purkinje Cells Is Critical for Inhibitory Transmission and Contributes to Locomotor Coordination^1^,^2^,^3^

**DOI:** 10.1523/ENEURO.0028-16.2016

**Published:** 2016-06-15

**Authors:** Negah Rahmati, Maria Fernanda Vinueza Veloz, Jie Xu, Sharon Barone, Nahuel Rodolfo Ben Hamida, Martijn Schonewille, Freek E. Hoebeek, Manoocher Soleimani, Chris I. De Zeeuw

**Affiliations:** 1 Department of Neuroscience, Erasmus MC, 3000 CA Rotterdam, The Netherlands; 2 Department of Medicine, University of Cincinnati, Cincinnati, OH 45267; 3 Netherlands Institute for Neuroscience, 1105 BA, Amsterdam, The Netherlands

**Keywords:** Chloride homeostasis, Chloride channel, GABAerg inhibition, Cerebellum, Purkinje cell, Locomotion

## Abstract

Chloride homeostasis determines the impact of inhibitory synaptic transmission and thereby mediates the excitability of neurons. Even though cerebellar Purkinje cells (PCs) receive a pronounced inhibitory GABAergic input from stellate and basket cells, the role of chloride homeostasis in these neurons is largely unknown. Here we studied at both the cellular and systems physiological level the function of a recently discovered chloride channel, SLC26A11 or kidney brain anion transporter (KBAT), which is prominently expressed in PCs. Using perforated patch clamp recordings of PCs, we found that a lack of KBAT channel in PC-specific KBAT KO mice (L7-KBAT KOs) induces a negative shift in the reversal potential of chloride as reflected in the GABA_A_-receptor-evoked currents, indicating a decrease in intracellular chloride concentration. Surprisingly, both *in vitro* and *in vivo* PCs in L7-KBAT KOs showed a significantly increased action potential firing frequency of simple spikes, which correlated with impaired motor performance on the Erasmus Ladder. Our findings support an important role for SLC26A11 in moderating chloride homeostasis and neuronal activity in the cerebellum.

## Significance Statement

The cerebellum plays an essential role in controlling performance and adaptation of motor function, predominantly through the action of Purkinje cells (PCs). In addition to their excitatory glutamatergic input and intrinsic pacemaking activity, the firing behavior of PCs is determined by their inhibitory GABAergic input. However, despite the established role of chloride homeostasis in inhibitory synaptic transmission, little is known about the identity of chloride channels or transporters that regulate intracellular chloride in PCs. Using genetically engineered mice and perforated patch-clamp recordings we found that SLC26A11, which is highly expressed by PCs, functions as a chloride channel and plays an important role in moderating chloride homeostasis in PCs and cerebellar function.

## Introduction

The cerebellum is well known for its role in controlling performance and adaptation of motor function. The cerebellar cortex ultimately exerts its effects through its sole output, the Purkinje cell (PC; De Zeeuw et al., 2011). The firing patterns of PCs are determined by their glutamatergic excitatory input from granule cells and inferior olivary neurons, their GABAergic inhibitory input from molecular layer interneurons, as well as by their intrinsic pacemaking activity, resulting largely from Na^+^, Ca^2+^, and K^+^ currents (Raman and Bean, 1999; Khaliq et al., 2003; Sausbier et al., 2004; Womack et al., 2004; Akemann and Knöpfel, 2006; Kalume et al., 2007; Aman and Raman, 2010; Gao et al., 2012b; Benton et al., 2013). Interestingly, Cl^−^ channels of PCs may also have a profound impact on cerebellar activity and motor behavior by influencing the strength of their GABAergic inputs (Seja et al., 2012).

Recently, we identified a new Cl^−^ channel in the brain, SLC26A11 [also called KBAT (kidney brain anion transporter)], which is prominently expressed in PCs (Rahmati et al., 2013). KBAT is a member of the SLC26 family of anion transporters. Mutations in human *SLC26* genes have been linked to several autosomal recessive disorders, including chondrodysplasias, chloride losing diarrhea (CLD), and Pendred syndrome (deafness and enlargement of the vestibular aqueduct), which are caused by mutations in SLC26A2, SLC26A3, and SLC26A4, respectively. Our studies in HEK293 cells showed that KBAT is a constitutively active Cl^−^ channel and is not regulated by intracellular Ca^2+^ or cAMP (Rahmati et al., 2013). Recent studies on cortical and hippocampal pyramidal cells indicated a critical role of KBAT in cell swelling after cytotoxic brain edema (Rungta et al., 2015). High activity of KBAT appears to result in a remarkable Cl^−^ entry into the cell following significant Na^+^ entry and a strong depolarization of the membrane potential. Thus, because Cl^−^ entry contributes to neuronal swellings (Rungta et al., 2015), KBAT may be a key player in brain edema and form a potential therapeutic target for treatment of cerebrovascular accidents.

To understand the role of KBAT at the cellular and systems level during normal operations in the healthy brain we generated and investigated mice with specific deletion of KBAT in PCs (L7-KBAT KO) using the Cre/loxP system. We hypothesized that if KBAT transfers Cl^−^ inside PCs, its deletion may cause a decrease in [Cl^−^]_i_ and a negative shift in the reversal potential of GABA (E_GABA_). Our findings show that the absence of KBAT in PCs indeed results in a negative shift of E_GABA_, alters PC firing rates and affects coordination of limb movements.

## Materials and Methods

### Generation of PC cell-specific Slc26a11 (KBAT)-deficient mice

**Figure 1.**
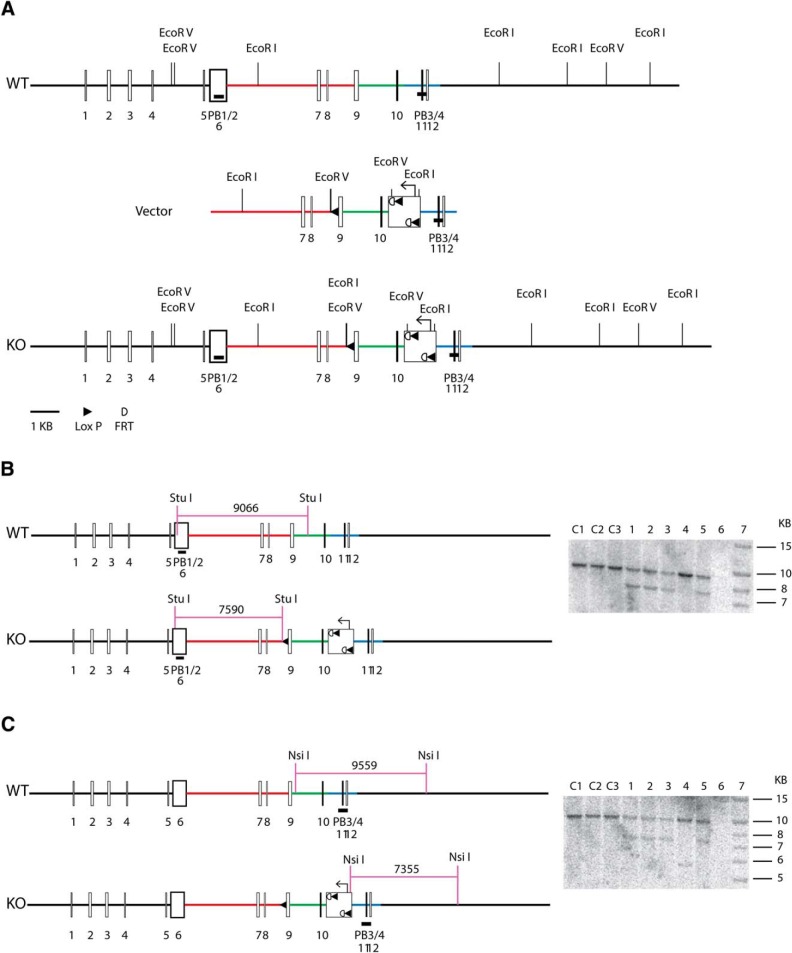
**Generation of Slc26a11 knock-out mice. *A***, A Slc26a11 conditional targeting vector containing the Slc26a11 genomic region was constructed to flox exons 9 and 10, flanked by long and short homology arms, respectively. The linearized targeting vector was electroporated into ES cells (for details see Materials and Methods). ***B***, DNA was digested with StuI, and was hybridized with a probe targeted against the 5′ external region. The expected sizes are indicated on the schematic and positive clones were further confirmed by Southern blot analysis (left). ***C***, DNA from the same clones was digested with NsiI, and was hybridized with a probe targeted against the 3′ internal region. The expected sizes are indicated on the schematic and positive clones were confirmed by Southern blotting (left).

To generate PC-specific KBAT-deficient animals, a Slc26a11 conditional targeting vector containing the Slc26a11 genomic region was constructed, in which the negative selective marker thymidine kinase gene, and the positive selective marker neomycin resistance (neo cassette) gene flanked by two Frt sites and two LoxP sites were introduced in relevant positions. The vector was designed to flox exons 9 and 10, flanked by 6.8 and 2.1 kb long and short homology arms, respectively (Fig. 1*A*). The arrangement of the LoxP sites in the knock-out construct was such that after the deletion of the neomycin resistance gene and the middle LoxP site, the remaining two LoxP sites flanked the 9 intron 5′ untranslated region, exons 9 and 10 and part of the intron 11 of the *Slc26a11* gene (the schematic of LoxP-Slc26a11 construct is shown in Fig. 1*A*). The linearized targeting vector was electroporated into BA1 (C57BL/6x129/SvEv) (Hybrid) ES cells as before. After dual selections by G418 and ganciclovir and screening by genomic PCR, several ES cells containing the KBAT conditional targeted allele were identified. The identity of ES cells was further confirmed by Southern blot analysis. DNA was digested with StuI, and was hybridized with a probe targeted against the 5′ external region. The expected sizes are indicated on the schematic and positive clones were further confirmed by Southern blotting analysis using an internal probe (Fig. 1*B*). DNA from the same clones was digested with NsiI, and was hybridized with a probe targeted against the 3′ internal region. The expected sizes are indicated on the schematic and positive clones were confirmed by Southern blotting (Fig. 1*C*). Two correctly targeted ES cell clones were used for blastocyst injection to generate chimeric mice, which were then crossed with WT C57BL/6 mice to obtain mice capable of germ line transmission of KBAT conditional knock-out gene. The tail DNA was analyzed by PCR. The PCR primers are A1: 5′-GGA GAA CAT CAA TTA CAT CCC ACT CC-3′; LAN1: 5′-CCA GAG GCC ACT TGT GTA GC-3′; LUNI: 5′-GCA TCG CCT TCT ATC GCC TTC TTG-3′; S26C3: 5′-GGT CTT GGT CTT GCC TTT CTA AGG-3′; SDL2: 5′-GGT CTT GGT CTT GCC TTT CTA AGG-3′. The animals were bred with Flp recombinase transgenic mice (obtained from the Gene Targeting Mouse Service Core at the University of Cincinnati, Cincinnati, OH) to obtain conditional KBAT KO mice with floxed allele lacking the neo cassette. These mice were crossed with wild-type C57BL/6 mice to remove Flp recombinase transgene in order to generate mice heterozygote for KBAT conditional knock-out gene (KBAT^+/flneo−flp−^), which are designated as KBAT^fl/fl^ or KBAT^+/fl^ mice. After elimination of the neomycin resistance and Flp recombinase genes, the resulting conditional KBAT-KO^Neo−/Flp−^ mice were backcrossed to C57BL/6 mice for >8 generations to obtain KBAT^Neo−/Flp−^ mice on C57BL/6 genetic background. These animals were mated with L7 promoter-driven Cre recombinase transgenic mice in order to disrupt *Slc26a11* gene expression in the PCs (Barski et al., 2000). The Cre-recombinase-mediated disruption of *Slc26a11* gene was confirmed by examining the genomic DNA. Briefly, isolated genomic DNA was amplified (94°C for 2 min, 35 cycles of 94°C 30 s, 61°C 30 s, 68°C 60 s, and then 68°C for 10 min) using S26C3 (5′-GGTCTTGGTCTTGCCTTTCTAAGG-3′) and SDL2 (5′-TTAGCGAGCGTCGACTTGGTAAG-3′) primers. The amplified DNA was examined for the presence of 419 bp wild-type (WT) and 351 bp (conditional KBAT-KO) products. The ablation of Slc26a11 in PCs was verified by Northern hybridization in the cerebellum (Fig. 1*D*) and immunofluorescence labeling using KBAT-specific antibodies (Fig. 1*E*). Mice of the following genotypes from both genders were used for the experiments: KBAT^-/fl^/Cre+ (also referred to asL7-KBAT KO or homozygous/+) and KBAT^-/fl^/Cre− (also referred to as homozygous/−), KBAT^+^/Cre+ (referred to as wild-type/+) and KBAT^+^/Cre− (also referred to as wild-type/−); the latter three groups were used as WT littermate controls. Animals received water and food *ad libitum*. All experimental procedures were approved by the institutional guidelines at Erasmus MC in Rotterdam and University of Cincinnati.

### RNA isolation and Northern blot analysis

Total cellular RNA was extracted from mouse cerebellum according to established methods, quantitated spectrophotometrically, and stored at −80 °C. Total RNA samples (30 μg /lane) were fractionated on a 1.2% agarose-formaldehyde gel, transferred to Magna NT nylon membranes, crosslinked by ultraviolet light, and baked. Hybridization was performed according to established methods. The membranes were washed, blotted dry, and exposed to a PhosphorImager screen (Molecular Dynamics). Slc26a11 variant-specific ^32^P-labeled cDNA fragments (Xu et al., 2011; Rahmati et al., 2013) were used as probes for Northern hybridizations. The band densities on Northern hybridization were quantitated by densitometry using ImageQuaNT software (Molecular Dynamics).

### Antibodies and Western blot analysis

KBAT-specific antibodies (Xu et al., 2011; Rahmati et al., 2013) were used for Western blot analysis. Briefly, microsomal membrane proteins were prepared from cerebellum of WT and KBAT KO mice. Thereafter, proteins were size-fractionated by SDS/PAGE (50 µg/lane) and transferred to nitrocellulose membrane. Western blot analyses were performed using anti-KBAT (Slc26a11) antibodies. Appropriate horseradish peroxidase-conjugated IgGs (Thermo Scientific) were used as secondary antibodies. The bands were visualized by chemiluminescence method (Invitrogen), and captured on light-sensitive imaging film (Denville Scientific).

### Immunofluorescent staining

Mice were transcardially perfused through the left ventricle with 4% paraformaldehyde (in 0.1 m phosphate buffer). Brains were removed, postfixed in the same fixative for 1 h at room temperature, and then placed in 10% sucrose (in 0.1 m phosphate buffer) at 4°C overnight. Brains were embedded in gelatin and then protected in 30% sucrose (in 0.1 m phosphate buffer) at 4°C overnight. Forty-micrometer-thick slices were cut with a freezing microtome and washed with tris-buffered saline (TBS), pH 7.6. Sections were incubated in 10 mm Na^+^-citrate at 80°C for 2 h. Next, sections were permeabilized and blocked in TBS containing 0.4% Triton X-100 and 10% normal horse serum at room temperature for 1 h. Thereafter, Rabbit polyclonal Slc26a11 (KBAT) antibodies (1:250) were applied to the sections at 4°C for 48 h. For nuclear staining, sections were treated with DAPI for 15 min. For visualization, green (FITC or AlexaFluor 488) fluorescent-conjugated secondary antibodies (Jackson ImmunoResearch) were used at 1:200 dilution. Sections were mounted, covered with Vectashield mounting medium for fluorescence (Vector Laboratories). Images were acquired on a confocal laser scanning microscope (LSM 700; Zeiss) with the same settings of laser power (405 and 488 nm excitation wavelength), optical and digital zoom, and filter for both L7-KBAT KO mice and WT controls, and optimized equally for contrast and brightness manually (Zen 2012 software, Zeiss).

### Slice preparation for electrophysiology

Sagittal slices of cerebellar vermis (250 mm) of P21 to P35 L7-KBAT KO and their WT littermate controls were prepared in ice-cold oxygenated “slicing” solution containing the following (in mm): 240 sucrose, 5 KCl, 1.25 Na_2_HPO_4_, 2 MgSO_4_, 1 CaCl_2_, 26 NaHCO_3_, and 10 d-glucose. Slices were transferred to artificial CSF (ACSF) containing the following (in mm): 124 NaCl, 2.5 KCl, 1.25 Na_2_HPO_4_, 2 MgSO_4_, 2 CaCl_2_, 26 NaHCO_3_, and 20 d-glucose, bubbled with 95% O_2_ and 5% CO_2_ for 30 min at 34°C. Sections were kept in ACSF in room temperature and used for recordings after 1 h recovery. PCs were visualized using an upright microscope (Axioskop 2 FS plus; Carl Zeiss) equipped with a 40× water-immersion objective. Data were collected using an EPC-10 amplifier (HEKA Electronics).

### Gramicidin-perforated patch-clamp recordings

To prevent disruptions of endogenous intracellular Cl^−^ concentration, a subset of recordings were performed using the perforated patch-clamp technique (Abe et al., 1994; Ebihara et al., 1995; Kyrozis and Reichling, 1995; Owens et al., 1996). Gramicidin stock (10 mg in 200 μl DMSO) was prepared and diluted to 50 µg/ml in the recording pipette solution containing the following (in mm): 140 KCl, 5 MgCl, 10 HEPES, 5 EGTA; osmolarity 290, pH 7.25–7.35, adjusted with KOH. Recording pipettes of 3–5 MΩ were tip-filled with gramicidin-free recording solution and backfilled with gramicidin-containing solution. PCs were approached with recording electrodes and when the resistance at the tip of the electrode surpassed 1 GΩ the command potential was set to −60 mV. Recordings were commenced once the gramicidin-evoked decrease in access resistance stabilized below 60 MΩ. Patch pipettes were also used for the pressure application (5 ms, 5 psi) of GABA_A_-agonist muscimol by a pneumatic pump (PV 800; World Precision Instruments). These pipettes were filled with ACSF including 100 µm muscimol and located near the presumed location of proximal dendrites. The bath solution (ACSF) contained 1 µm TTX to block action potentials. The reversal potential of GABA_A_-current (E_GABA_) was extracted from the currents evoked by puff application of muscimol while clamping the cell at various membrane potentials for 1 s. All membrane potentials subsequently were corrected for the voltage drop across the series resistance:

(1) $${\text{V}}_{\text{corr}}={\text{V}}_{\text{com}}-{\text{I}}_{\text{clamp}}\times {\text{R}}_{\text{s}},$$

in which *V_com_*is the command potential, *I_clamp_*is the clamp current, and *R*_S_ is the series resistance. Assuming that E_GABA_ is equal to E_Cl_^−^, [Cl^−^]_i_ was calculated using Nernst equation (Eq. 2; Kaila and Voipio, 1987; Brumback and Staley, 2008; Seja et al., 2012), where *R* is the gas constant (8.315 J·mol^−1^ ·K^−1^), T is the temperature (in Kelvin), *F* is the Faraday’s constant (96.487 C·mol^−1^), [Cl^−^]_i_ is the intracellular concentration of Cl^−^ and [Cl^−^]_o_ is the extracellular concentration of Cl.

(2) $${\text{E}}_{\text{Cl}}=-\frac{\text{RT}}{\text{F}}\times \frac{{\text{ln[Cl]}}_{\text{o}}}{{\text{[Cl]}}_{\text{i}}}.$$

It should be noted that the [Cl^−^]_i_ in general may vary substantially between recorded neurons, even in the same region of the brain (Barmashenko et al., 2011), potentially due to their position in the slice and their volume (Glykys et al., 2014).

### Cell-attached recordings of PC and molecular layer interneurons

PCs and molecular layer interneurons (MLIs) from lobule V of the vermis were recorded at 34 ± 1ºC in cell-attached mode using pipettes filled with ACSF. The recordings of PCs were performed in the presence of 10 μm NBQX and 10 μm APV in order to block all AMPA- and NMDA-mediated synaptic inputs, respectively. In a subset of experiments, inhibitory synaptic currents were additionally blocked by bath application of 100 μm picrotoxin.

### Whole-cell recordings of PC

PCs from lobule V of the vermis were recorded in whole-cell current-clamp mode using pipettes filled with the following (in mm): 124 K-gluconate, 9 KCl, 10 KOH, 4 NaCl, 10 HEPES, 28.5 sucrose, 4 Na_2_ATP, and 0.4 Na_3_GTP, pH 7.25–7.35, osmolarity 295. Ionotropic excitatory and inhibitory inputs to PCs were blocked by bath application of 10 μm NBQX, 10 μm APV, and 100 μm picrotoxin. PCs that required more than −700 pA current injection to maintain the holding potential at −65 mV were discarded. We injected 800 ms current steps ranging from −100 to 1000 pA with 100 pA increments. Action potential firing rates were measured over the entire traces and compared for the various levels of current injection. Action potential properties (threshold, peak amplitude, afterhyperpolarization, and half-width) were evaluated using the first action potential generated by each PC. The afterhyperpolarization is calculated as the relative difference with the action potential threshold. The liquid junction potential (calculated to be −10.2 mV) was not corrected.

### Spontaneous IPSCs recordings of PCs

PCs from lobule V of the vermis were recorded in whole-cell voltage-clamp mode using pipettes filled with the following (in mm): 140 CsCl, 4 NaCl, 0.5 CaCl_2_, 10 HEPES, 5 EGTA, and 2 MgATP. The use of an internal solution that contains high concentrations of Cl^−^ facilitates the detection of small events, but reverses the polarity (IPSCs occurs as negative, inward currents; Wulff et al., 2009; Gao et al., 2014). EPSCs were blocked by bath application of the ionotropic glutamate receptor blockers NBQX (10 µm) and APV (10 µm). Each cell was recorded for 2 min at −75 mV. Series resistance was compensated online to reduce the residual <10 MΩ. Data were digitized at 20 KHz and analyzed using miniAnalysis software (Synaptosoft).

### *In vivo* extracellular recordings of PCs

Extracellular recordings of PCs were performed in adult mice (2–4-month-old L7-KBAT KO and WT control). Pedestal surgeries and craniotomies were performed as described previously (Rahmati et al., 2014). After surgery, mice were allowed to recover for at least 3 days before one day of habituation to the setup and subsequent electrophysiological recordings. During the recordings, mice were head-fixed but able to freely walk on a wheel. Extracellular recordings were performed using filamented glass pipettes (Harvard Apparatus) of 4–8 MΩ (1.5 mm outer diameter × 0.86 mm inner diameter) filled with 2 m NaCl that were positioned by a motorized micromanipulator (SM5, Luigs & Neumann). All recordings were performed in the vermal lobules at a minimal depth of 500 µm from pial surface. Recordings were identified as single-unit PC recordings if the pause in simple spike firing following each complex spike was at least 8 ms (Zhou et al., 2014). During the recordings, the openings in the skull were covered with saline. Recordings were subsequently amplified, filtered, and digitized using MultiClamp 700B and Digidata 1440A devices (Axon CNS, Molecular Devices).The firing frequency of simple spikes and complex spikes, as well as their regularity (CV and CV2) were analyzed offline and compared between L7-KBAT KO mice and the controls using the MATLAB-based program SpikeTrain (Neurasmus, Erasmus MC Holding).

### Erasmus Ladder tests

Detailed analysis of gait patterns of L7-KBAT KO mice and their littermate controls were performed and compared by using Erasmus Ladder paradigm as described previously (Vinueza Veloz et al., 2014). Briefly, each mouse had to perform one daily session during 8 d. Each daily session consisted of 72 trials during which the mouse had to walk back and forth between two shelter boxes. Each shelter box is equipped with a LED spotlight in the roof and two pressurized air outlets in the back. Sensory stimuli (light and air) serve to control the moment of departure of the mice. The ladder has 37 rungs on each side, and each rung can be displaced vertically following a command from the control system. Even-numbered rungs on one side and odd-numbered rungs on the other were elevated by 6 mm, thereby creating a left/right alternating pattern. All rungs are equipped with custom-made pressure sensors that are continuously monitored. The baseline locomotion of mice was assessed in the first four sessions (non-perturbed sessions). During the last four sessions, we tested locomotion adaptation by challenging the mouse to deal with the appearance of an obstacle, which was preceded by a tone 200 ms prior to its occurrence (perturbed sessions). The obstacle was induced randomly by elevating one of the lower rungs (Vinueza Veloz et al., 2014). In all sessions, the step size was determined as the distance between two consecutive touches. Likewise, step time was defined as the time between the onsets of two consecutive touches. The size and time of steps per session were compared between L7-KBAT KO mice and wild-type controls.

### Compensatory eye movements

Vestibulo-ocular reflexes were measured as previously described (Wulff et al., 2009; Schonewille et al., 2010; Seja et al., 2012). Mice were head-fixed by a surgically implanted pedestal and positioned in the center of a turntable, which was surrounded by a cylindrical drum with a random dotted pattern. Mice were subjected to sinusoidal rotation of the drum in light [optokinetic reflex (OKR)] or of the table in dark [vestibulo-ocular reflex (VOR)] or light [visually enhanced VOR (VVOR)] to test their baseline motor behavior. On subsequent days, adaptation of VOR was induced. On the first day of VOR adaptation training, both table and drum were rotated in phase at the same frequency (0.6 Hz) and amplitude (5°). The next days, the drum amplitude is increased to 7.5° on day 2 and 10° on days 3–5. In between the 1 h recording sessions, the mice were kept in the dark (overnight). After calibrating, averaging, and fitting the eye movements, drum and/or table stimuli traces, the eye movement gain was calculated as the ratio between pupil and stimulus velocity and the eye movement phase as their timing difference in degrees.

### Drugs

All chemicals for electrophysiological experiments were purchased from Tocris Bioscience and Sigma-Aldrich, prepared as stock solutions and stored before use as aliquots at the manufacturers’ recommended temperature and conditions.

### Statistics

*In vitro* and *in vivo* electrophysiological results were tested for significant differences using an unpaired, two-tailed Student’s *t* test, unless stated otherwise. Behavioral data obtained with the Erasmus Ladder paradigm and compensatory eye movements were analyzed using SPSS (IBM) and tested for statistically significant differences by two-way, repeated-measures ANOVA. *p* Values ≤0.05 were considered statistically significant. The experimenters were blind to the genotypes of the mice until data analyses were completed. Data are represented as mean ± SEM.

## Results

### Slc26a11 (KBAT) is selectively deleted from cerebellar PCs

**Figure 2.**
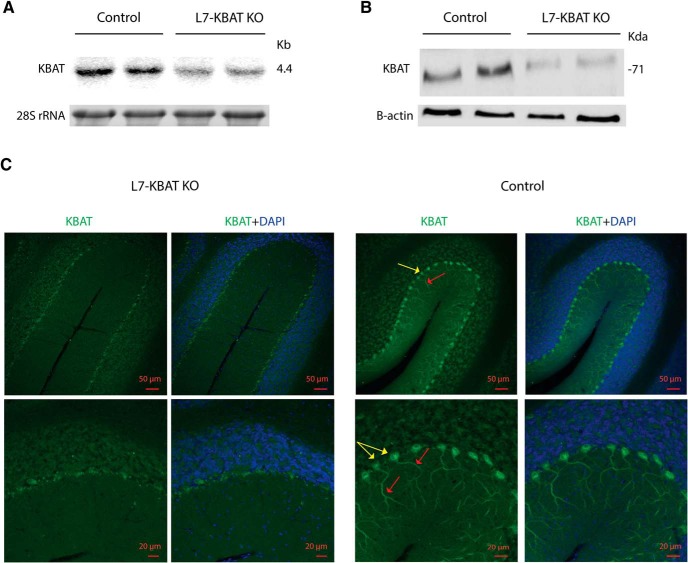
**KBAT is specifically deleted from PCs. *A***, Northern blot analysis of cerebellar RNA showing a significant reduction in KBAT mRNA expression levels in KBAT knock-out mice. ***B***, Western blot analysis of cerebellar proteins showing a significant reduction in KBAT protein abundance in KBAT knock-out mice. ***C***, Immunofluorescent staining of KBAT in cerebellar tissues of wild-type mice shows a strong labeling of KBAT in cell body and dendrites of PCs (top), whereas KBAT is deleted from PCs of L7-KBAT KO mice. KBAT localization is shown in green. Blue represents DAPI nuclear staining. Yellow arrows indicate PC cell bodies and red arrows their dendrites.

A neo-cassette replaced 6.8 kb of the long arm of exon 9 and 2.1 Kb of the short arm of exon 10 (Fig. 1*B*,*C*). Two correctly targeted ES cell clones were used for blastocyst injection to generate chimeric mice, which were then crossed with WT C57BL/6 mice to obtain mice capable of germ line transmission of Slc26a11 (KBAT) conditional knock-out gene. The identity of ES cells was further confirmed by Southern blot analysis (Fig. 1*B*,*C*). Slc26a11^lox/lox^ mice were crossed with L7 promotor-driven Cre recombinase transgenic mice (Barski et al., 2000) in order to specifically disrupt Slc26a11 (KBAT) expression in PCs (L7-KBAT KO). All offspring survived normally, showed no gross behavioral phenotype, and were indistinguishable from wild-type littermates in their home cage. Northern and Western blot experiments on the cerebellar vermis showed significant reduction of KBAT mRNA and protein expression in L7-KBAT KO mice (Fig. 2*A*,*B*). Immunofluorescent staining of sagittal brain slices confirmed the deletion of KBAT from cerebellar PCs (Fig. 2*C*).

### Deletion of Slc26a11 (KBAT) affects the GABA reversal potential, but not the resting membrane potential in PCs

**Figure 3.**
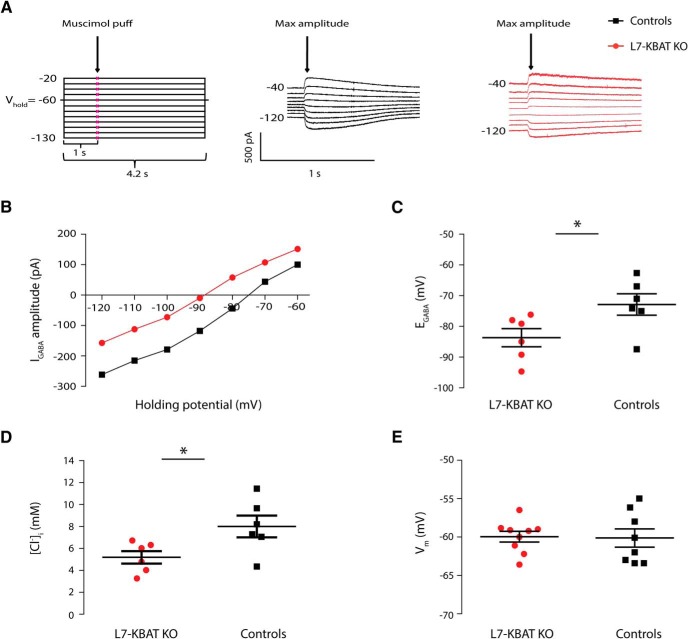
**KBAT regulates E_GABA_. *A***, Left, The schematic representation of the protocol used for calculation of E_GABA_ by perforated patch-clamp recording to avoid disruption of the [Cl^−^]_i_. Right, Raw traces of a L7-KBAT KO PC and a control PC. ***B***, *I*/*V* curve of the same representative PCs in ***A***. Each data point indicates the maximum current amplitude evoked by puff application of muscimol to a L7-KBAT KO PC and a control PC when clamped at various holding potentials (ranging from −120 to −60 mV with 10 mV intercept). ***C***, Comparing the reversal potential of GABA obtained from PCs of L7-KBAT KO mice shows a significant negative shift compared to littermate controls (*p* = 0.03, *n* = 6 per group). ***D***, The estimated intracellular concentration of Cl^−^ is significantly reduced in L7-KBAT KO mice (*p* = 0.03, *n* = 6 per group). ***E***, The membrane potential of PCs is not significantly different between L7-KBAT mice and controls (*n* = 8 for control and *n* = 9 for L7-KBAT KO PCs; *p* = 0.8).

To examine the role of KBAT in PC Cl^−^ homeostasis, we measured GABA-induced Cl^−^ currents using gramicidin-perforated patch-clamp recordings. To do so, we applied the GABA_A_R agonist muscimol to proximal dendrites of PCs. The reversal potential of this GABA_A_-current (E_GABA_) was extracted from the currents evoked while clamping the cell at different holding potentials (Fig. 3*A*,*B*). The E_GABA_ in L7-KBAT KO PCs was significantly more hyperpolarized than in control PCs (L7-KBAT KO: −83.70 ± 2.95 mV; controls: −72.88 ± 3.46 mV, *n* = 6 per group, *p* = 0.03; Fig. 3*C*). Using these data, the estimated [Cl^−^]_i_ was measured and found to be significantly different between the knock-out and control mice (Fig. 3*D*). On average [Cl]_i_ was 35% lower in PCs lacking KBAT (L7-KBAT KO: 5.18 ± 0.56 mm; controls: 7.99 ± 0.98 mm, *n* = 6 per group, *p* = 0.03). However, the resting membrane potential was not significantly different in KO PCs (L7-KBAT KO: −59.95 ± 0.69 mV, *n* = 9; control: −60.13 ± 1.19 mV, *n* = 8, *p* = 0.8; Fig. 3*E*).

### Deletion of KBAT altered the frequency of spontaneous inhibitory postsynaptic currents

**Figure 4.**
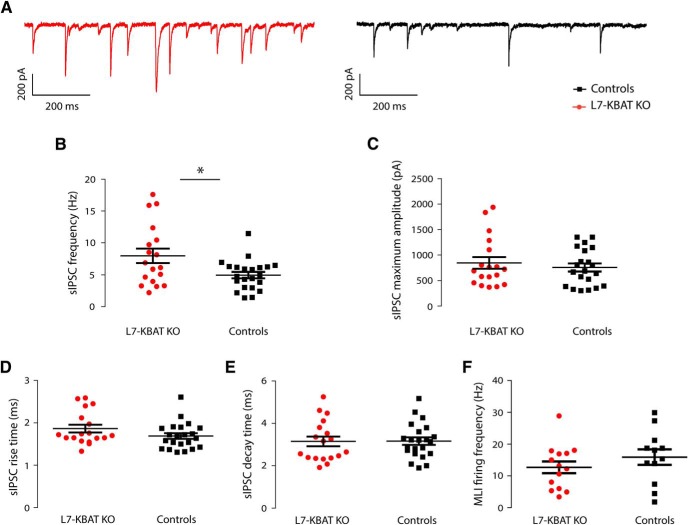
**sIPSCs in PCs. *A***, Representative traces of sIPSCs of a L7-KBAT KO PC (right) and a control PC (left). ***B***, Frequency of sIPSCs is significantly higher in PCs of L7-KBAT KO mice (*n* = 22 control PC and 18 L7-KBAT KO PC; *p* = 0.01). ***C***, Comparing the maximum amplitude of sIPSCs did not show a significant difference between the two groups (*p* = 0.51). ***D***, ***E***, No significant difference was detected in neither the rise time of sIPSCs (P = 0.12) nor the decay time (*p* = 0.95). ***F***, *In vitro* cell-attached recordings of MLIs did not show a significant deference in the spiking activity of presynaptic interneurons. Each panel shows individual data points with mean values ± SEM.

In principle, the more negative E_GABA_ results in a higher driving force (DF) for Cl^−^ to go inside the cell. To study whether this affects the GABAergic inhibition of PCs lacking KBAT, in the next series of experiments we recorded the frequency and amplitude of spontaneous IPSCs (sIPSCs; Fig. 4*A*). These experiments revealed that the frequency of sIPSCs was significantly higher in L7-KBAT KO cells (L7-KBAT KO: 7.95 ± 1.13, *n* = 18; control: 4.95 ± 0.49, *n* = 22, *p* = 0.01; Fig. 4*B*). Comparing the maximum amplitude of sIPSCs between knock-out and control cells did not show a significant difference (L7-KBAT KO: 842.5 ± 115.0, *n* = 18; control: 754.3 ± 77.5, *n* = 21, *p* = 0.51; Fig. 4*C*). Modifications of intracellular ionic composition of PCs may also affect the kinetics of the inhibitory currents. However, the rise and decay time of sIPSCs showed no significant differences (Fig. 4*D*,*E*; *p* values > 0.05). To investigate whether the rise in frequency of sIPSCs is due to increased MLI action potential firing, we recorded their activity in cell-attached configuration (similar to the PC recordings reported in Fig. 4*A*). The firing frequency of MLIs did not show a significant change in the KO mice (L7-KBAT KO: 12.70 ± 1.86, *n* = 14; control: 15.93 ± 2.44, *n* = 12; *p* = 0.29; Fig. 4*F*).

### KBAT contributes to spontaneous firing of PCs

**Figure 5.**
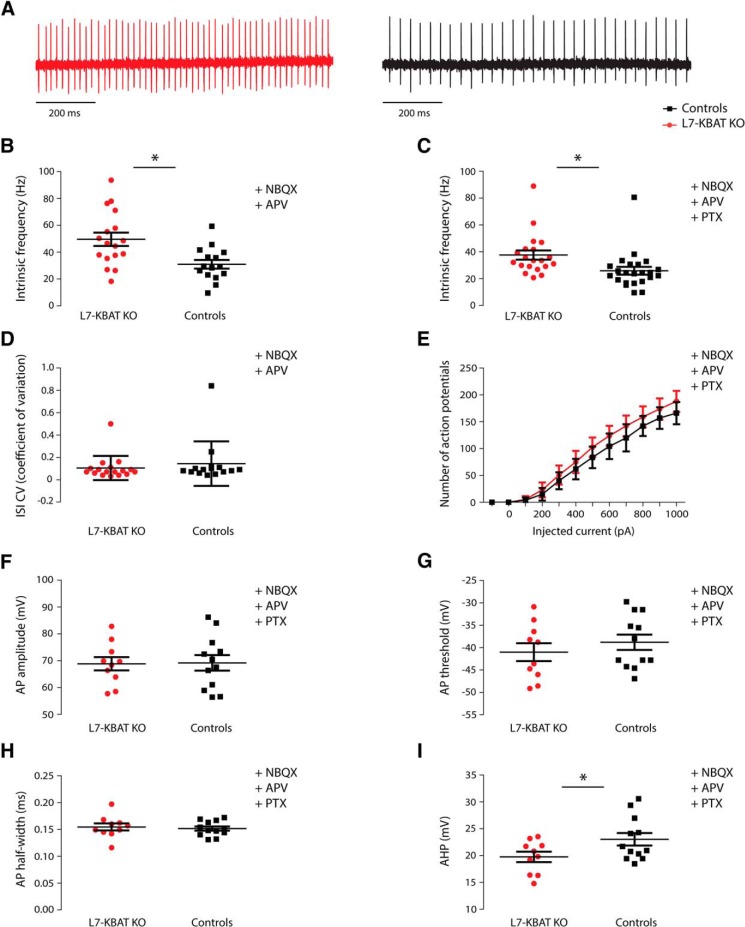
**Spontaneous and evoked action potential firing in PCs. *A***, Representative traces of intrinsic firing activity of a L7-KBAT KO PC (left) and a control PC (right). ***B***, Spontaneous firing frequency was significantly higher in KO PCs compared to the controls when the inhibitory input was present (*n* = 15 control PC and 17 L7-KBAT KO PC; *P* = 0.004). ***C***, Firing frequency of PCs remained significantly higher when both inhibitory and excitatory inputs were blocked (*n* = 23 control PC and 20 L7-KBAT KO PC; *p* = 0.01). ***D***, The regularity of firing between the two groups did not show a significant difference (*p* = 0.9). ***E***, The excitability of PCs was compared by injecting depolarizing currents to PC in steps of 100 pA and did not show a significant difference between the groups (repeated-measures ANOVA, *p* = 0.2, *n* = 12 per group). ***F*–*H***, Action potential amplitude, threshold and half-width were not significantly different (*n* = 12 per group; *p* > 0.05). ***I***, The amplitude of AHP was significantly smaller in KO PCs compared with controls (*n* = 12 per group; *p* = 0.04).

In the next series of experiments, we hypothesized that if GABA mediated inhibition is stronger in PCs of KBAT knock-out mice their action potential firing patterns may be affected. To test that, we first recorded the spontaneous action potential firing of PCs (Fig. 5*A*), which in our conditions is known to be under control of GABAergic molecular layer interneurons (Häusser and Clark, 1997). L7-KBAT KO PCs showed a significantly increased firing frequency compared to the controls (L7-KBAT KO: 49.59 ± 4.96, *n* = 17; controls: 30.98 ± 3.24, *n* = 15 and, *p* = 0.004; Fig. 5*B*). The regularity of spontaneous firing was not significantly different between the groups (*p* = 0.9; Fig. 5*D*). To unravel whether the difference in spontaneous firing frequency is due to changes in the intrinsic pacemaking activity of PCs or in the inhibitory synaptic input, we next repeated these recordings in a separate set of experiments in the presence of the GABA_A_R-blocker picrotoxin. In a similar fashion, PCs from L7-KBAT KO mice showed a significantly higher firing frequency (L7-KBAT KO: 37.59 ± 3.46, *n* = 20; controls: 25.90 ± 2.91, *n* = 23, *p* = 0.01; Fig. 5*C*). Considering the higher firing frequency of PCs in L7-KBAT KO mice, one possibility is that PCs are more excitable in these mouse models. To test that, we performed whole-cell patch clamp recordings in current clamp mode while injecting 100 pA steps of depolarizing current. The resulting action potential firing showed no significant difference in frequency (*n* = 12 per group, *p* = 0.2; Fig. 5*E*). Moreover, the action potential amplitude, threshold, and half-width were also not different between the groups (Fig. 5*F*–*H*). However, PCs of L7-KBAT KO mice showed a smaller afterhyperpolarization (AHP) amplitude (L7-KBAT KO: 19.76 ± 0.96; controls: 23.03 ± 1.17, *p* = 0.04; (Fig. 5*I*), whereas the input resistance was not significantly different between the groups (*p* = 0.94; Table 1).

**Table 1.** Passive properties of PCs

	RMP, mV	*I*_holding_, pA	*R*_s_, MΩ	*R*_in_, MΩ	*C*_m_, pF	τ_m_, ms
L7-KBAT KO	−56.3 ± 1.6	−262 ± 42	10.5 ± 0.7	59.4 ± 4.5	180 ± 10	10.6 ± 0.9
Controls	−55.5 ± 1.6	-308 ± 90	9.89 ± 0.39	60.0 ± 6.9	196 ± 19	11.3 ± 1.0
*p* value	0.72	0.26	0.44	0.94	0.47	0.66

RMP: Resting membrane potential; *I*_holding_: holding current at −60 mV, *R*_s_: series resistance; *R*_in_: input resistance; *C*_m_: membrane capacitance; τ_m_: membrane time constant. *N* = 6 per group.

### Deletion of KBAT changes the firing pattern of PCs *in vivo*

**Figure 6.**
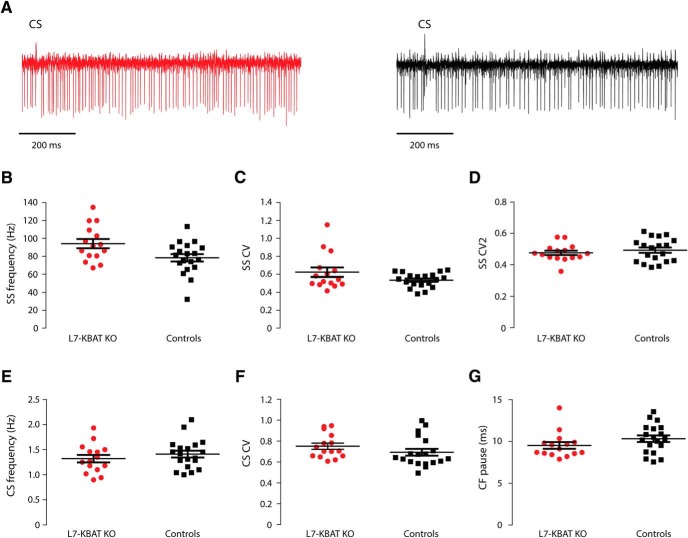
***In vivo* PC firing. *A***, Single-unit traces of a L7-KBAT KO PC (left) and a control PC (right). ***B***, The frequency of simple spikes (SS) was significantly higher in PCs lacking KBAT (*n* = 19 control PC and 15 L7-KBAT KO PC; *p* = 0.02). ***C***, The regularity of simple spike firing (CV) did not show a significant difference between the groups (*p* = 0.09). ***D***, The regularity of interspike intervals (CV2) of simple spikes was not affected in L7-KBAT KO mice (*p* = 0.4). ***E***, The frequency of complex spikes was not changed in the knock-out cells (*p* = 0.3). ***F***, The regularity of complex spike firing (CV) was not significantly different between the groups (*p* = 0.2). ***G***, The minimal CF pause of the recorded cells was not significantly different (*p* = 0.1).

The experiments performed in slices indicated that L7-KBAT KO PCs showed an increased PC firing rate. To investigate whether this increased action potential firing prevailed in the living animal, we next performed extracellular recordings in awake, head-fixed mice. These *in vivo* recordings (Fig. 6*A*) displayed a significant increase in simple spike firing frequency of PCs in L7-KBAT KO mice compared to wild-type controls (L7-KBAT KO: 94.13 ± 5.12, *n* = 15; controls: 78.32 ± 4.12, *n* = 19, *p* = 0.02; Fig. 6*B*). The regularity of simple spikes (CV) was not significantly affected in KO PCs (L7-KBAT KO: 0.62 ± 0.05, *n* = 15; controls: 0.53 ± 0.01, *n* = 19, *p* = 0.09; Fig. 6*B*). The regularity of interspike intervals (CV2) of simple spikes also did not achieve statistical significance in KO PCs (L7-KBAT KO: 0.47 ± 0.01, *n* = 15; controls: 0.49 ± 0.01, *n* = 19, *p* = 0.4; Fig. 6*C*). PC complex spike firing, which represents the activity of inferior olive neurons, also showed no significant differences in either frequency (L7-KBAT KO: 1.32 ± 0.07, *n* = 15; controls: 1.41 ± 0.06, *n* = 19, *p* = 0.3) or regularity (Fig. 6*E*–*G*; *p* values > 0.05). The climbing fiber pause did not exhibit a statistically significant difference between the KBAT KO PCs and the controls (L7-KBAT KO: 9.51 ± 0.39, *n* = 15; controls: 10.32 ± 0.39, *n* = 19, *p* = 0.1). These data suggest that deletion of KBAT from PCs changes the firing frequency of these cells both *in vitro* and *in vivo* without affecting their regularity.

### PC-specific KBAT KO mice show altered locomotion

**Figure 7.**
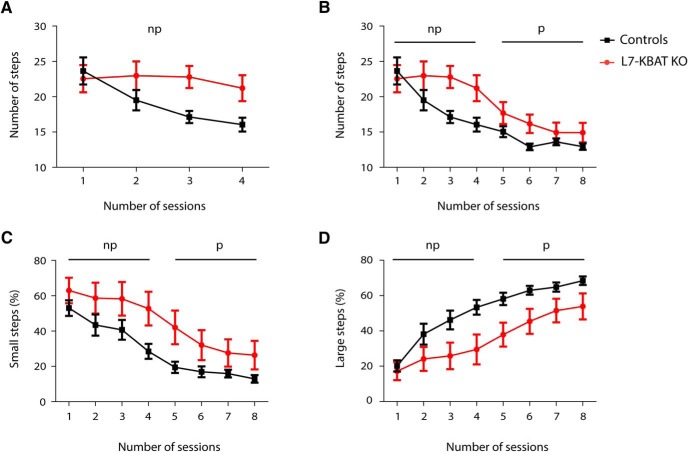
**L7-KBAT KO mice show smaller step size compared with littermate controls. *A***, Comparison of average number of steps during baseline (non-perturbed) sessions shows significant higher number of steps per session in L7-KBAT knock-out mice compared to the controls (*p* = 0.02, *n* = 10 per group). ***B***, Average number of steps per session during both perturbed and non-perturbed sessions shows that the difference in the number of steps remains significantly different during all sessions (*p* = 0.02). ***C***, L7-KBAT knock-out mice show higher percentage of small steps in all sessions compared to the wild-type control mice (*p* = 0.02). ***D***, Percentage of large steps is significantly lower in L7-KBAT KO mice (*p* = 0.01). Non-perturbed (np) and perturbed (p) sessions are indicated in each panel.

It was previously shown that aberrant PC firing induces impaired locomotion (Vinueza Veloz et al., 2014). Therefore, we subjected L7-KBAT KO mice and their littermate controls to the Erasmus Ladder behavioral paradigm. Erasmus Ladder enables us to perform a detailed analysis of locomotor activity of mice by quantification of their step size and step time while they walk on a horizontal ladder (Vinueza Veloz et al., 2014). Analysis of the first baseline (non-perturbed) sessions showed that the average number of steps is significantly higher in L7-KBAT KO mice (Fig. 7*A*; *p* = 0.02). Although the knock-out mice began to decrease the number of steps in later sessions, the difference in number of steps remained significant in all sessions (Fig. 7*B*; *p* = 0.02). Throughout all sessions L7-KBAT KO mice showed a higher percentage of small steps and lower percentage of large steps (Figs. 7*C*,*D*). The average step time was not significantly different between the groups (data not shown).

### Deletion of KBAT does not affect the vestibulo-ocular reflex

**Figure 8.**
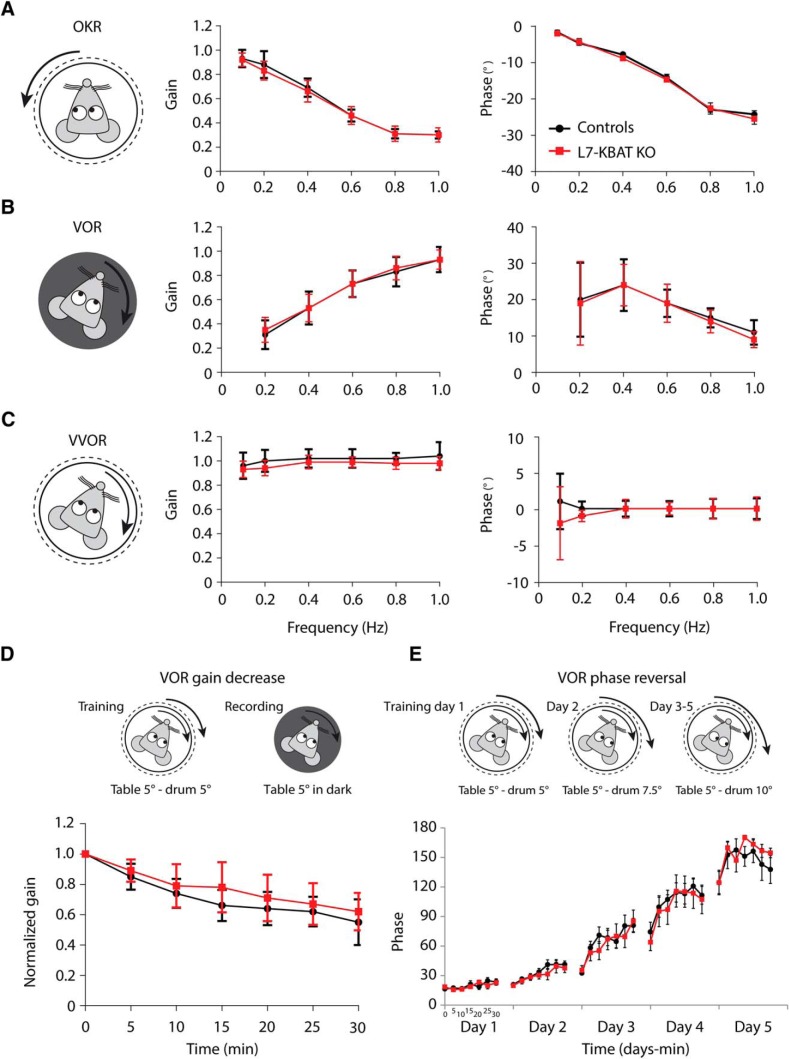
**Eye movement tests were not affected in L7-KBAT mice. *A*–*C***, Compensatory eye movements in adult control (*n* = 12) and L7-KBAT KO (*n* = 12) were compared. Mice were subjected to visual (OKR) and/or vestibular stimulation (in dark, VOR; in light, VVOR) and gain (ratio of eye to stimulus velocity) and phase (difference in degrees between eye and stimulus) were calculated. None of these parameters were detected to be different between knock-out and control mice (*p* values > 0.05). ***D***, VOR gain-decrease learning was not impaired in L7-KBAT KO mice compared with controls (*p* = 0.1). ***E***, Likewise, VOR phase reversal training did not show a difference in motor learning and consolidation between the L7-KBAT KO mice and the control mice (*p* = 0.5).

To elaborate the behavioral characterization of the L7-KBAT KO mice we also tested the compensatory eye movements using optical and vestibular stimuli, which are known as highly sensitive behavioral measurements for cerebellar motor performance and motor learning. Previous studies on many PC knock-out mouse models with aberrant simple spike firing have shown impaired motor performance and/or motor learning when subjected to the compensatory eye movement paradigm (De Zeeuw et al., 2011; De Zeeuw and Ten Brinke, 2015). Thus, to find out whether an increase in simple spike activity can also be detrimental we subjected the L7-KBAT KO mice to the same tasks. Recordings of baseline motor performance by measuring the gain and phase of OKR, VOR, and VVOR did not show significant differences between the L7-KBAT KO mice and their WT controls (Figs. 8*A*–*C*). We next subjected the mice to VOR gain-decrease and phase-reversal training paradigms to examine the motor leaning between L7-KBAT KO mice and the controls. Here too, neither VOR gain-decrease nor phase-reversal tests revealed significantly different responses in L7-KBAT KO mice compared with control littermates (Figs. 8*D*,*E*).

## Discussion

Here we identified Slc26a11 (KBAT) as a novel determinant of [Cl^−^]_i_. Our results indicate that deleting KBAT induces a reduction in [Cl^−^]_I_, as well as a negative shift in E_GABA_. These findings are in line with our previous studies on HEK293 cells, which highlighted the role of KBAT as a Cl^−^ accumulator (Rahmati et al., 2013). Studies on other knock-out models of Cl^−^ accumulators have also shown a shift of E_GABA_ to more hyperpolarized potentials as a result of diminished [Cl^−^]_i_ (Sung et al., 2000; Gonzalez-Islas et al., 2009). Interestingly, these changes in L7-KBAT KO mice were accompanied by increased PC action potential firing both *in vitro* and *in vivo*, despite the absence of a significant effect on the resting membrane potential. Typically, a negative shift of E_GABA_ does not increase the action potential firing rate, unless the change in E_GABA_ is accompanied with modifications of other ion channels and the ionic composition of the intracellular environment. Impaired motor coordination together with altered PC action potential firing point towards a link between Cl^−^ homeostasis in PCs and their role in cerebellar function.

The impaired locomotor activity of mutant mice lacking KBAT when subjected to the Erasmus Ladder paradigm suggests an important role of KBAT in cerebellar motor behavior (Vinueza Veloz et al., 2014). L7-KBAT KO mice showed a significant increase in the number of small steps during walking on the ladder. A higher number of small steps can be considered as one of the major and most sensitive signs of locomotor dysfunction on the Eramus Ladder (Vinueza Veloz et al., 2014). So far, all mutant mouse models with altered PC activity have shown a smaller step size on the ladder, whereas this phenotype was not always accompanied with obvious signs of other motor abnormalities or ataxia (Vinueza Veloz et al., 2014). The behavioral phenotypes of L7-KBAT KO mice on the Erasmus Ladder were similar to those obtained from knock-out mice with PC-specific deletion of the γ2 subunit of GABA_A_R (L7-γ2 KO). Whether the MLI-PC input is more important for controlling the amplitude of movements rather than timing needs to be further investigated by other behavioral paradigms with a stronger requirement for proper timing, such as eyeblink conditioning (De Zeeuw and Ten Brinke, 2015). Possibly, there are different learning rules between PCs engaged in zebrin-positive and zebrin-negative zones (De Zeeuw and Ten Brinke, 2015). Indeed, we found a significant difference between the knock-out mice and their controls on the Erasmus Ladder, which is subject to control by zebrin-negative zones, such as those in lobule V (Hoogland et al., 2015), but no difference was detected during performance or adaptation of compensatory eye movements, which is a highly sensitive cerebellar task for the zebrin-positive regions. To date, an increased firing frequency of PCs has so far hardly been shown to affect performance and learning of a cerebellar behavior in the eye-movement domain (De Zeeuw et al., 2011; Gao et al., 2012a).

The negative shift of E_GABA_ in mutant cells while the resting membrane potential (*V*_m_) is intact raises the possibility that GABA_A_ receptors that are expressed by PCs have a higher tendency to transfer Cl^−^ inside the cell and thereby generate a stronger inhibitory impact on PCs. According to our studies, PCs of mutant mice lacking KBAT show a higher frequency of sIPSCs. The spontaneous firing frequency of MLIs was recorded to investigate the possibility of an increased activity of MLIs and their input to PCs. These experiments did not reveal a significant difference between the L7-KBAT KO and control mice. Although we did not find an evidence for enhanced presynaptic spiking, it is worth noting that the majority of granule cells in slice preparation do not fire action potentials and thus do not activate MLIs. Therefore, future studies should elucidate to what extent GC-MLI input of L7-KBAT KO mice is increased *in vivo.* In addition, it is plausible that deletion of KBAT from PCs may induce changes in presynaptic mechanisms, including vesicle filling and vesicle turnover.

KBAT knock-out mice form an atypical manifestation of Cl^−^ modification in neurons in several ways. First, the intrinsic firing frequency of their PCs was significantly increased both at the *in vitro* and *in vivo* level, despite an enhanced frequency of IPSCs. Second, the complex spike frequency was normal, despite an increase in simple spike firing frequency. One could have expected a higher complex spike frequency, when simple spike frequency is increased, as reverberating activity in the nucleo-olivary loop normally mediates such homeostatic effects (Chen et al., 2010; Zhou et al., 2014). Finally, the level of regularity of simple spike firing in KBAT knock-out was not significantly altered, despite the fact that an altered GABAergic input to PCs normally induces a change in the regularity of their firing pattern (Wulff et al., 2009; Seja et al., 2012). Together, these data point towards induction of a myriad of compensatory mechanisms that may have altered the intrinsic properties of both PCs and other cells in the olivocerebellar system (De Zeeuw et al., 2011).

Deletion of KBAT may have changed PC activity by multiple mechanisms. It is possible that other Cl^−^ channels/transporters could compensate for the reduction in [Cl^−^]_i_ subsequent to KBAT inactivation. For example, the activation of NKCC1 or inhibition of KCC2 can potentially improve the [Cl^−^]_i_ toward normal levels, albeit along with an increase in intracellular concentration of Na^+^ and/or K^+^. Such a compensatory response suggests that KBAT inactivation could modify the transmembrane gradient of other ions as well. Whether KBAT deletion can alter the activity of Cl^−^/HCO_3_^−^ exchangers, such as AE3 (from SLC4 family of bicarbonate transporters) or SLC26A7 (from SLC26 family of anion transporters) remains to be determined.

The higher firing frequency of PCs raises the possibility that KBAT might also have influenced Na^+^ currents as a result of altered [Cl^−^]_i_, but we could not detect any difference in the threshold or amplitude of the action potentials. Surprisingly, the amplitude of AHP was significantly reduced in L7-KBAT KO PCs, which may be the reason for the increased action potential firing. The amplitude of AHP amplitude is mostly regulated by Ca^2+^-activated K^+^ channels (BK and SK channels; Sah, 1996; Faber and Sah, 2002). Indeed, inhibition of SK channels can result in a higher frequency of PCs (Womack et al., 2004), further emphasizing the possibility of up- and/or down regulation of particular Ca^2+^, K^+^, and/or Ca^2+^-activated K^+^ channels in L7-KBAT KO mice.

It is important to also consider the possibility that Cl^−^ channels form physical or functional complexes with other channels and/or transporters. For instance, a Ca^2+^-activated Cl^−^ channel (Ano3) in rat dorsal root ganglia (DRG) showed physical and functional interaction with a Na^+^-activated K^+^ channel (Slack; Huang et al., 2013). Deletion of Ano3 caused diminished expression of Slack and subsequent hyperexcitability of DRG cells (Huang et al., 2013). There are additional examples of physical and/or functional interactions of Cl^−^ channels with certain ion channels/transporters in other tissues. Studies on mouse kidney have shown the functional collaboration of ClC-Kb (a voltage-gated Cl^−^ channel from ClC family) with NKCC2 and ROMK (Kir1.1), a K^+^ channel. Inactivating mutations of ClC-Kb lead to the inactivation of NKCC2 and ROMK channel and cause Bartter syndrome, a rare heterogeneous autosomal recessive nephropathy, characterized by reduced salt reabsorption in the kidney thick ascending limb of Henle, resulting in renal salt wasting and hypokalemic metabolic alkalosis (Konrad et al., 2000). Likewise, it is plausible that deletion of KBAT could result in the dysregulation of molecules/transporters that physically or functionally interact with it. Studies aimed at identifying the binding partners of KBAT (ie, via yeast 2 hybrid expression system) or ascertaining the impact of KBAT deletion on compensatory or maladaptive mechanisms (via RNA-Seq) will provide a deeper insight into the role of KBAT in cerebellar function.

## Synthesis

The decision was a result of the Reviewing Editor Uwe Heinemann and the peer reviewers coming together and discussing their recommendations until a consensus was reached. A fact-based synthesis statement explaining their decision and outlining what is needed to prepare a revision is listed below. The following reviewer(s) agreed to reveal their identity: Ray Turner

The reviewers see merit in this paper as it addresses the possible role of a new player in regulation of E (IPSP) beyond regulation by KCC2 or NKCC1, bicarbonate as regulated by Carboanhydrase and the relation between bicarbonate and Cl due to effects of AE3 (anion exchanger 3). Hence the study is timely and interesting. The most important fact is they have identified a new factor controlling Cl gradients in Purkinje cells despite all the years of work on this cell type. However there are still problems with the Ms which must be addressed.

The following points should be addressed:

1. The authors state

"The negative shift of EGABA in mutant cells while the resting membrane potential (Vm) is intact raises the possibility that GABAA receptors that are expressed by PCs have a higher tendency to transfer Cl- inside the cell and thereby generate a stronger inhibitory impact on PCs. Indeed, PCs of mutant mice lacking KBAT showed a higher frequency of spontaneous inhibitory post-synaptic currents (sIPSCs). "

The observation that frequency of IPSCs goes up is interesting but cannot be explained by an increase in driving force. That said, if the authors would like to explore the impact of altered E Cl on spontaneous IPSCs, then these experiments would need to be carried out with gramicidin patches and not be based solely on whole cell recordings with high Cl solution inside the pipette

2. An alternative explanation for the increase in IPSC frequency might be a secondary increase in presynaptic properties.

We suggest to state that presynaptic changes may be involved. Since this would require more in depth studies and possible explanations might only be mentioned in the discussion. These could include alterations in vesicle filling, changes in vesicle turnover, secondary changes in network properties and the like but they can at least exclude firing increase in one type of neurons.

If they want really to exclude any network alternation they might decide to record mIPSC frequency in presence of TTX.

We feel that the authors can just modify their text by just reporting their finding including that they found no evidence that enhanced presynaptic spiking is responsible and leave it at that.

3. An increase in IPSC frequency will not lower intracellular Cl concentration but rather increase it if IPSPs are hyperpolarizing. Only if IPSPs are depolarizing lowering of intracellular Cl may result. Is there evidence that interneurons in cerebellum cause depolarizing IPSCs onto pyramidal cells?

4. The authors indicate data regarding spontaneous firing in MLIs, this data should be included in the figures

5. The authors report no differences in input resistance, but don't report the actual values. This data should be included. It would be helpful to show a table containing basic parametric data from control cells and mutant cells to show that recordings are comparable. These would include Rinput, Cm, Rseries, holding current, RMP, etc.

6. As mentioned in the prior the prior reviews, talking about "trending toward significance" regarding CV values and relying on one-tailed T-tests is not sound scientific methodology. Given the very small effect size and lack of significance the authors should either back away from this claim. If the authors insist on the claim they would have to do more experiments and support their conclusions by a power analysis.

7. perhaps one can show a less cartoon type picture of mice but this is not mandatory but would help in general in discussion with animal protectionists.
